# Educational Response to a Student with Psychosis at the Secondary Level: A Non-Experimental Single-Case Study

**DOI:** 10.3390/ejihpe10040076

**Published:** 2020-11-24

**Authors:** Juan Pedro Martínez-Ramón, Inmaculada Méndez, Cecilia Ruiz Esteban

**Affiliations:** Department of Evolutionary and Educational Psychology, University of Murcia, 30100 Murcia, Spain; juanpedromartinezramon@um.es (J.P.M.-R.); cruiz@um.es (C.R.E.)

**Keywords:** educational intervention, health promotion, psychosis, quality of life, non-experimental study

## Abstract

Students with psychosis in school within the ordinary education system are a reality in the classroom. To study their correct adaptation at school, it is necessary to consider numerous factors such as the personal characteristics of the student, environmental variables, educational measures put in place as well as emotional and cognitive aspects. The aim of this research was to monitor the teaching–learning process of a student diagnosed with psychosis and enrolled in a public school at the secondary level in the ordinary modality with support during an academic year, with the usual resources provided by a guidance department to assess the impact of the educational measures and plans on his emotional and academic fields. This was a single case study in which both qualitative and quantitative information was collected (*N* = 1). The participant was a student with special needs at the secondary level. An analysis of the results of psychometric tests, plan for diversity, observational analysis, academic file, scholastic history, and multiple interviews were carried out. The findings show how the educational curriculum can be adapted to improve the competences of a student with psychosis by encouraging an increase in social abilities and potential cognitive abilities through the counseling department. The conclusions of this research can provide a guideline for comparison of different educational systems, paying greater attention to the development of emotional aspects, and opting for inclusive measures. In this line, this study shows that students with psychosis can share classrooms and studies with their peers, thus fulfilling the principle of educational inclusion.

## 1. Introduction

Psychosis encompasses a wide range of symptoms and characteristics, such as thought disorders with effects on speech and writing, hallucinations, and delusional beliefs [1,2]. The psychotic symptomatology in adolescents is diverse and covers both cognitive and behavioral aspects [3]. Cognitive behavioral therapies have proven effective in improving the adaptation of individuals with psychosis [4]. However, many other variables can affect the development and intervention in psychosis. So, genetic factors are involved in this disorder [5,6], and social factors are also of paramount importance. For instance, the result of a systematic review showed that family involvement is essential in the intervention with people with psychosis [7]. Other factors, such as consumption habits, also have to be considered [8].

Besides, previous stress levels seem to be partly linked the emergence of the disorder in adolescence [9]. Regarding the educational field, the presence of psychosis can adversely affect academic outcomes [10]. In general terms, adolescence is a time of change for any student and these changes can be difficult. This period is more complicated when a mental illness is part of the equation. For this reason, the educational system must be prepared for it. Another factor that must be considered is the student’s intrinsic motivation, as promoting intrinsic motivation in an individual with psychosis may be useful to improve psychological functioning [11]. The self-motivating ability is essential for explaining the learning strategies of the student [12]. One aspect that has not been studied widely is the effect of emotional education in the traditional educational system on students. This competence is key for decision making and establishing functional social relationships with other people [13]. 

In line with the previous paragraph, students with psychosis often have difficulties in managing their emotions. This includes the ability to recognize their own emotions as well as those of others. This is a basic aspect in human interaction and its absence has a negative impact on their social adaptation and on school coexistence [14]. 

One of the closely related concepts of emotional adjustment is alexithymia, which consists of the inability to manage emotions, to recognize one’s own emotional expressions and those of others, so that people with high levels of alexithymia have greater difficulties in relationships. People with psychosis have difficulties in the emotional sphere and may have high levels of alexithymia [15].

A recent study confirmed the economic benefits of early intervention due to health care system benefits and savings [16]. The benefits of detecting and applying appropriate measures exceed the perceived personal benefits and are also socially valued, justifying for promotion of this type of study. Evidence-based practice is a useful method for sharing positive education experience [17]. The educational system has a key role in both early detection and intervention [9]. Also, individuals with psychosis show a lower performance in cognitive tests [18], so those students with lower intellectual capacity have less elaboration capacity [19]. In this field, educational psychologists are prepared to cope with these tasks.

Educational psychologists must respond to the needs of the environment, which involves considering different viewpoints through a systemic approach. The educational psychologist is not only responsible for assessing the situation, but also for investigations and providing advice [20]. In practice, educational intervention, coupled with prevention, are the main tasks to analyze through the prism of educational psychology. However, interventions are not applied for other issues such as absenteeism or special educational needs [21]. For this reason, it is necessary to promote these types of variables in investigations. 

Another reason to investigate the current situation of students with psychosis in the educational system is the stigma that exists in this respect. Today, there is still a misconception about the potential of such a student in the school. It is erroneously stated that students with psychosis have little potential to learn how to adapt to their environment, manage their emotions, develop their social skills to solve the difficulties that may arise from human interaction, among other aspects [22].

The justification for describing how the adaptation process of a student with psychosis is outlined in secondary education responds to the fact that it is possible to use the measures that the educational system possesses to facilitate coexistence as much as possible and to develop the emotional intelligence of the subject. In order to publicize these measures and ensure that the educational community benefits from them, measures are implemented that are based on evidence-based practice [23,24]. Accordingly, it is necessary to share the professional experiences that one has in order to encourage other people to put them into practice and bet on an ordinary schooling of the students with psychosis, whenever possible.

### Objectives and Hypothesis

Our main aim was to monitor the teaching–learning process of a student diagnosed with psychosis and enrolled in a public school at the secondary level during an academic year to assess the impact of the educational measures and plans on his emotional and academic fields. This goal was subdivided into other specific goals: (1) Study his adaptation to the academic environment (through the objective recording of academic variables such as grades and absences); (2) to examine the progress of his emotional development by detecting any difficulties that may exist through the analysis of levels of alexithymia and in his perception of the enjoyment of life; and (3) to analyze the measures of attention to diversity and orientation implemented in the educational center. 

The initial hypotheses were:

**Hypothesis 1** **(H1).** *The student’s academic performance is expected to improve as the academic year progresses, since he will have been exposed to educational measures for longer*.

**Hypothesis 2** **(H2).** *The ability to recognize his emotions and his capacity to enjoy life is expected to increase as the academic year progresses—therefore, the more time the student is at the center, the better the adaptation of student*.

**Hypothesis 3** **(H3).** *It is expected that there will be a number of plans and strategies based on educational standards*.

## 2. Materials and Methods 

### 2.1. Participant

The subject was a male student aged 14 and enrolled in the first semester in a public secondary school in Spain and was selected incidentally. At the time of writing, he was repeating that semester. His level of competence was located at first course of secondary. The school provides secondary and high education and there are 26 more students in the subject’s class. Until recently, they had a diagnosis of specific language disorder. Afterward, the center was informed that the student had psychosis as diagnosed by the mental health center. The subject is receiving medication. The educational center was located in the southeast of Spain and there were no relevant conflicts at the time of the evaluation, since this could have affected the levels of psychotic symptomatology as outlined in a recent systematic review and meta-analysis [25]. The student had an intellectual capacity limit (IQ = 75), which was measured using the Wechsler Intelligence Scale for Children, fourth edition (WISC-IV). The results of the other measures are listed in Table 1. 

### 2.2. Instruments

The instruments and documents used for evaluation included:

- Questionnaire for the analysis of academic and professional orientation in secondary education (COAPES) [26]. It consists of 36 items with open and closed answers. It assesses the functioning and organization of the school in which the student was enrolled. The internal consistency index of the questionnaire reached 0.894 measured by Cronbach’s alpha.
- Toronto’s Alexithymia Scale (TAS-20) [27], adapted to Spanish in the version of [28]. It is a psychometric instrument composed of 20 items arranged on a Likert-type scale ranging from 1 to 5 where 1 means *Totally disagree* and 5 means *Totally agree*. It consists of three factors: “Difficulty in identifying feelings” composed of items 1, 3, 6, 7, 9, 13, and 14 (example of item: “I have physical sensations that even doctors do not understand”); “Difficulty in describing feelings” composed of items 2, 4, 11, 12, and 17, (example of item: “I have physical sensations that even doctors do not understand”); and “Pattern of externally oriented thinking” composed of items 5, 8, 10, 15, 16, 18, 19, and 20, (example of item: “I prefer to let things happen, rather than analyze why they happened”). The test reached a Cronbach’s alpha consistency index of 0.078 [28].
- Savoring Beliefs Inventory (SBI), Spanish version of [29] of the original scale of [30] It consists of 24 items expressed by means of a Likert-type scale that goes from 0 = *Nothing* to 4 = *A lot* or *Extremely*, distributed in three factors: “Anticipation” refers to the ability to be able to visualize a pleasant event or situation before it happens and is composed of items 1, 4, 7, 9, 10, and 13 (e.g., “I don’t like to think in advance about good times before they happen”); “Enjoy a present moment” is the capacity to be able to find pleasure in carrying out an activity at present and is composed of items 2, 3, 5, 11, 15, 17, 18, 19, 21, and 23 (e.g., “I enjoy remembering the happy moments I have experienced in my past”); and “Reminiscence” which is the ability to be able to evoke a positive event that has happened before, being composed of items 6, 8, 10, 12, 14, 16, 20, 22, and 24 (e.g., “When it comes to enjoyment I am “my worst enemy”). The overall internal consistency measured through Cronbach’s alpha was 0.91.
- Qualitative and quantitative analysis of the psycho-pedagogical report. This report included an assessment of his level of curricular competence, learning style, and a test of intellectual capacity. In particular, the Wechsler Intelligence Scale for Children, fourth edition, WISC-IV [31] is composed of the following global indexes: Verbal comprehension index (VCI), perceptual reasoning index (PRI), working memory index (WMI), and processing speed index (PSI). Using all these indexes, intelligence quotient (IQ) was obtained.
- Analysis of the plan for diversity and other related measures that consist of the center’s plan to address the educational needs of students. We conducted observational analysis through the guard’s break to collect useful and spontaneous information about his behavior. We analyzed his academic file, scholastic history, and other documents provided by the family about his personal life. Finally, we conducted interviews with the student, his family, and teachers during the course, with some meetings being spontaneous and others previously scheduled. These interviews dealt with relationships, human interactions, classroom rules, school coexistence, social skills, the tutorial action plan, classroom methodologies, and so on. Both the tools and the procedure identified are part of the functions that define the profile of the educational counselor.

### 2.3. Procedure

During the process of assessment, we maintained continuous communication with the family, teachers, and student. The family authorized the center to contact other institutions such as associations and health care centers. We obtained family consent for conducting this research by developing a document based on The Declaration of Helsinki and its ethical principles including being informed, and participating voluntarily and anonymously. The phases are described in Table 2. The duration of the sessions and/or actions varied depending on available time for direct attention and subject response. Each action was considered a work session. Notably, the school year is structured into three quarters.

The first phase was more focused on collecting information. The second phase focused on the adjustment of the measures proposed in the first phase through educational programs and plans, ensuring the proper functioning of the same, and seeking personal and material resources in the area. In the third phase, we analyzed the achievements, assessed the possible actions, and proposed new measures for next year, by focusing on the resources required and the most appropriate academic route for this student.

With regard to the subject’s stationary state, at the beginning, high levels of alexithymia were identified in him, low levels in the openness to enjoy life (as will be developed in the results) as well as features considered habitual in this situation: unstructured language (both oral and written), irrational fears and hallucinations (this last symptom controlled through pharmacological treatment). This research focused on analyzing the academic aspects (performance and absenteeism), emotional aspects (alexithymia and enjoyment of life), and the resources related to attention to diversity that were put in place during the course. 

The study period was a school year in Spain, i.e., from September to June. Information was collected continuously, and psychometric tests were administered at three times (one time per term).

### 2.4. Data Analysis

A case study non-experimental (*N* = 1) was conducted. Qualitative and quantitative information were collected through the instruments outlined above. Following [32], the single case design of this research shares features of an intrinsic and instrumental case study, which means that the case is interesting in itself because of the characteristics, and at the same time, to obtain a global vision of a problem. The selection of such a design allows for flexibility in the study steps by using an eclectic approach. The model, which is closest to the phases implemented in this study, was the basic A-B model by making a few nuances. In phase A, a baseline was drawn up describing the current situation of the student before starting the treatment (in this case “treatment” was understood as the application of the plan of attention to diversity in that academic year). In phase B certain parameters were measured again, analyzing their evolution, and comparing them with the baseline. Due to the fact that a lot of qualitative information was collected continuously and that phase B is based on a global plan in which it was not possible to control many of the variables (maturity aspects, social and family environment, teachers’ methodology, etc.), the model is considered to be non-experimental.

The qualitative analysis was mainly concerned with the extraction of information from psycho-pedagogical reports, file documents, and school plans. The quantitative analysis included a descriptive analysis of central tendency and dispersion measures. The statistical package SPSS (version 24) and Excel were used.

## 3. Results

### 3.1. Academic Variables

Concerning the first objective, the subject was enrolled in 11 subjects provided in Table 3. Marks are expressed according to the Spanish educational system. The minimum score is 1 and the maximum is 10. Tutoring is one of the subjects, although this is not evaluable. Through this weekly class, students use group dynamics and group activities to learn values, emotional intelligence, and study techniques. 

**Figure 1:**
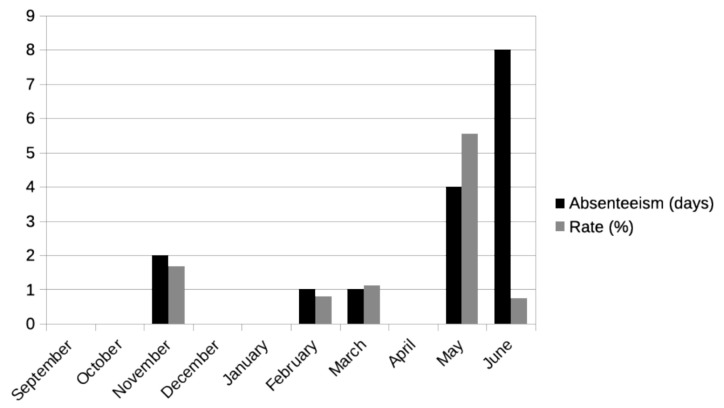
Absenteeism per month. Source: Own elaboration.

Concerning social abilities, the student presented difficulties with school integration. The subject had improved since last year, potentially because it was his second year at the school. According to the analysis of the psycho-pedagogical report, psychomotor skills and autonomy were appropriate. Both linguistic and social skills were affected. He had several problems understanding certain social situations, which caused misunderstandings and conflicts. Social skills and emotional intelligence were learned in the school for improving student strategies. His habits were apparently healthy, and he was not suspected of taking drugs. At specific moments, the student showed grossly disorganized behavior, especially manifested through speech. At those times, the specialist teacher helped the student relax, which fostered a more orderly linguistic structure. The student had dyslalias in spontaneous speech, which was also disorganized. Teachers claimed during the systematic interviews that the behavior of the student had improved. Regarding class assistance, the student’s rate of absence within the course was close to 1%. Figure 1 shows the student’s absence rate depending on the month. Each month had approximately 20 school days. In November, for example, he missed two days of school, which was equivalent to a rate of less than 2% absenteeism. The level was higher in recent months.

### 3.2. Emotional Development

**Figure 2:**
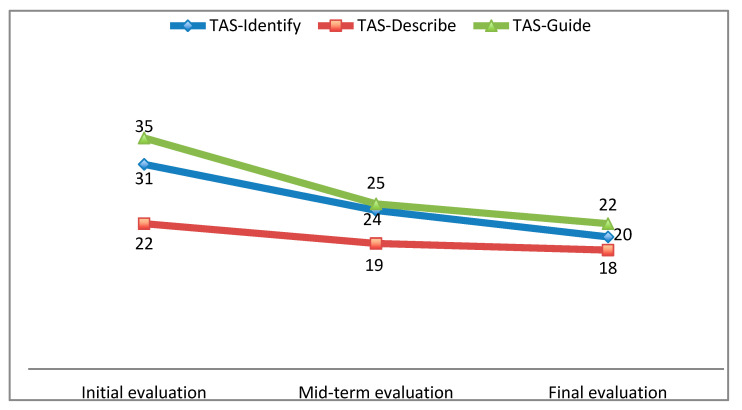
Evolution of alexithymia levels. Source: Own elaboration.

Regarding the second objective, Figure 2 shows the evolution of the levels of alexithymia (TAS-20) in the three factors that make it up, measured in three moments of the academic year: TAS-Identify (“Difficulty in identifying feelings”), TAS-Describe (“Difficulty in describing feelings”), and TAS-Guide (“Pattern of externally oriented thinking”). The first measurement took place during the initial assessment of the course (baseline), the second measurement during the month of February, and the third measurement in the month of June with an approximate distance of four months between the measurements. As can be seen, there was a progressive decrease in the levels of alexithymia more rapidly during the first four months.

**Figure 3:**
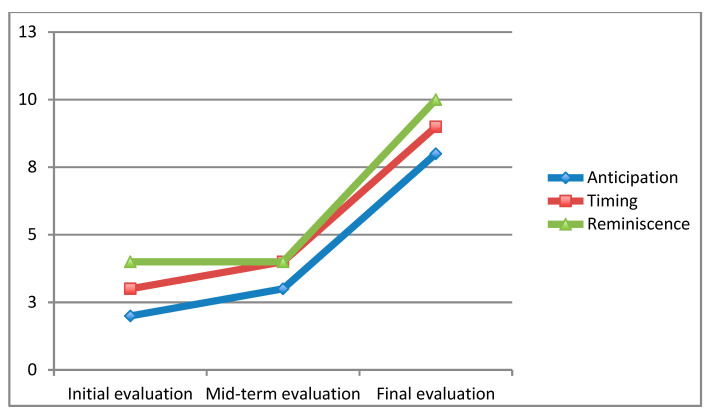
Evolution of the perception of enjoyment of life. Source: Own elaboration.

Figure 3 shows an increase in the subject’s ability to perceive life more positively (SBI) as the academic year progresses in the three factors: reminiscence, timing, and anticipation. There was a greater increase at the end of the course. 

### 3.3. Measures of Attention to Diversity

With regard to the third objective, generally speaking, specific human resources are needed to keep pace. Specifically, he was assigned a therapeutic pedagogy specialist teacher and a specialist teacher in hearing and language.

Regarding effective measures, natural environments and peer interaction had to be promoted as well as functional and generalizable vocabulary in familiar contexts. Particularly, vocabulary had to be used that allowed the student to positively develop social skills and conflict resolution. This aspect was promoted, and the student’s behavior improved during this course in comparison with the last one. 

The plan for diversity identified general, ordinary, and specific measures for attending to the needs of the student. In Table 4, different types of actions that are conducted in the center are detailed. Those measures appropriate for a student with psychosis were selected.

Firstly, general performance included the whole educational community, regardless of whether useful for a particular group. Secondly, ordinary measures were addressed to a group of students (including the student with psychosis). These measures could be applied considering a specific student but with the intention to apply to an entire classroom. Thirdly, specific measures were applied when the above measures had been implemented and had not yielded the expected results. These measures referred to methodologies and programs for a very specific type of student or need (e.g., psychosis). The main legislation is shown in Table 5. Other programs highlight the tutoring plan to encourage students to develop in other aspects such as emotional intelligence, social skills, values, and study strategies. The academic and career guidance programs were also relevant and aimed to have students reflect on their future. 

The analysis of the educational center through the COAPES questionnaire [26] reflects that in the educational center there is a multidisciplinary approach to the attention to the diversity and orientation of the students. The objectives of the academic and professional orientation of the students are adapted to their needs, the participation and collaboration of the family is facilitated, information and training is provided to students with special needs, a guidance council is issued at the end of each academic year during secondary education, individual interviews are carried out, and a report is prepared at the end of the course. However, more specific objectives need to be designed and made explicit within the guidance plan.

## 4. Discussion

The main aim of this study was to “monitor the teaching–learning process of a student diagnosed with psychosis and enrolled in a public school at the secondary level during an academic year”. With regard to the initial hypotheses, no improvement was observed in academic outcomes as the academic year proceeded so the hypothesis (H1) was rejected. The implementation of measures to deal with diversity during the course did not result in an improvement in student marks. Besides, the student was repeating the course, so higher grades were expected. Scores in subjects may not be the best criteria for assessing the academic learning process in this case and so new criteria could be considered. The disease progression could have been responsible for these marks as psychosis affects the learning process [3].

Concerning H2, it is confirmed. Without assuming causality with other variables, there was an improvement in his emotional adjustment. It is worth noting that the levels found in alexithymia in our research were higher than in the normal population; similarly, the subject’s enjoyment of life was lower than in the population, but relatively similar to the clinical sample in a previous study [29]. It is inferred that students with psychosis will obtain high levels of alexithymia in line with previous studies [15,41,42]. 

Regarding H3, it is confirmed that there are measures in place to address the diversity of students with psychosis in the education system. Analyzing the qualitative assessments of the educational agents involved in the student’s learning process, they agree that the student’s behavior had improved. This positively affected the climate in the classroom too. Notably, coordination among all professionals was fundamental. Note that students with psychosis have more difficulty adapting to the environment [19], which can lead to a longer period required to obtain positive results from programs for diversity. For this reason, it is expected that the longer the student is in the education system, the better the adaptation will be. However, we have to consider that the absence rate was higher in the last two months of the course. Thus, adaptation and absenteeism must be interpreted as two different concepts. 

The analysis of educational measures that were applied showed that the education system has different methods of addressing the diversity of students and the measures were applied throughout the course effectively. For this, teacher training is essential [43]. Not all teachers are aware of these measures. Secondary schools have a guidance department that can provide this training and guide the most appropriate academic option. A specific role of the educational counselor is to complete a psycho-pedagogical report. During the student’s course, the psycho-pedagogical report was written to identify the personal resources the subject needed, by using the above instruments. An individualized work plan was developed for the student. All teachers were coordinated through the guidance department and head of studies. The updating of the special educational needs of the student shows that it is necessary to promote cooperative work and socialization processes, provide aid while performing certain tasks, prioritize the development of language skills, and foster meaningful and functional activities. In summary, collaboration is essential. As such, it is necessary to involve educators in the educational measures because the more involved they are, the better the results achieved [9]. So, another recommendation that emerges from the analysis is that it is suitable to promote coordination between family members and teachers as well as the relevant services. This cooperation must exist from the beginning, before the first signs of the disorder arise, as psychosis could be related to exposure to previous stress levels [10]. The family reported large amounts of stress in his schooling due to an alleged case of bullying in previous courses.

On another front, the fact of having a student with clinical symptoms in school justifies the need to include knowledge derived from educational psychology in the training of professionals in guidance departments. Maybe, the lack of time is one of the main problems when managing cases like the present one. More instruments could be used to obtain more information and data. However, this is a difficult task due to the large number of cases, activities, different professional profiles, and functions. Hence, defining the roles of each educational agent is necessary to improve the system [20].

The information provided by teachers and the family indicated that the first year had been more difficult. The initial adjustment was complicated. A better mutual understanding may have influenced this improvement. When the teachers knew the needs of the student and the family, the school resources and possibilities, there was an improvement in relations.

To improve early detection and intervention of students with psychotic symptoms, it is necessary to promote awareness campaigns so the main warning signs are known to enable as early detection as possible [9]. In this case, the recommendation after completion of the psycho-pedagogical assessment was to continue studying in an ordinary school with specific human resources to promote the student’s integration.

With respect to the familial analysis and student habits, drugs were not detected. This was important to know as some experts claim that drug consumption is related to mental disease [8]. Also, both the instruments and procedures used in this investigation are typical of those used by a guidance counselors in the country, so this procedure can easily be replicated by other researchers in their daily lives to understand how their students are learning and to reflect on the measures they are applying. Sharing that information will be extremely useful to know what measures are working and which are not. Our findings could help researchers compare different countries, their methodologies, and educational systems.

Concerning the limitations of this study, the lack of time of the guidance counselor is one of the most important factors affecting the process and the ability to examine the case. Besides, family factors should be added into the analysis by examining the relationship between the members. So, it is necessary to further research the role the family [7]. More instruments and other kinds of analyses could be used. Aspects such as teacher assertiveness [44] or teacher stress [45] may have an influence on students and, for this reason, these aspects could be included in future research. The results could also have been affected by the social climate of the school and other coexistence measures aimed at combating bullying [46,47]. Eventually, it is necessary to take into account that it is not possible to confirm a causal relationship between the variables in this study design. 

In relation to applicability, this research illustrates that students with psychosis can benefit from schooling in an ordinary center, also at the secondary school stage, and that the diversity plan is a dynamic institutional document that can be used on students with psychosis, implementing measures to address diversity. Furthermore, considering the educational regulations allows for comparisons to be made in other studies with the rules and laws governing other regions and countries.

With regard to future research directions, a longitudinal study of these kinds of cases would be essential for understanding the adaptation of the individual to the environment. Similar studies could help identify the kind of measures that could be more effective with students diagnosed with psychosis in the classroom. In this regard, promoting evidence-based practice is a valid tool to share educational experiences among teachers and other experts. Future studies could review the influence of the teaching and learning process [47], creativity [19], intrinsic motivation [11,12], anxiety [4], and other healthy lifestyle habits that could be considered relevant, such as sleep quality [48,49].

## 5. Conclusions

To conclude, during the course of the course and as the plan of attention to diversity designed for the educational center has been applied, an improvement in the emotional processes in the student with psychosis has been observed. The student has reduced his levels of alexithymia and has improved his perception of vital aspects. This fact is interpreted as a greater capacity of adaptation to the school environment and a better personal functioning. This study also outlines the measures available for teachers and educational psychologists to intervene with special needs students. It should be noted that while the diversity plan can be associated with increased emotional functioning, it is not possible to claim the existence of cause and effect. Regarding policy implications, we consider it necessary that the development of this type of plan is prescriptive in the training of future teachers as well as the study of special educational needs during the university stage.

## Figures and Tables

**Table 1:** Scores obtained on the WISC-IV (*p* < 0.05).

Index	Sum of Scores	Composite Scores	Percentile
VCI	18	78	7
PRI	27	93	33
WMI	15	85	15
PSI	6	62	0.6
IQ scores	66	75 (IQ)	5

Note: VCI, Verbal Comprehension Index; PRI, Perceptual Reasoning Index; WMI, Working Memory Index; PSI, Processing Speed Index. Source: Own elaboration.

**Table 2:** Phases.

Phase	Actions	Descriptions
Phase 1, First quarter	Case analysis	Initial collection of information on the subject from the educational center
	Interview with family	Social and family analysis
	Interview with student	First contact. Obtained information about the main aspects: behavior, spontaneous language, etc.
	Initial evaluation	Before starting with 100% of the course content, the secondary school conducts an initial assessment to evaluate the initial level of the student and provide resources that are needed
	Design of plans and programs	Designing tutorial action plan, program of academic and professional guidance, and plan for diversity, considering the collected information
	Interview with teachers and head of studies	Collecting information about learning style, beginning of course, and peer relationships
	Interview with specialists	Tracking student progress and making decisions about the number of hours of specific support received and the material used
	Contact with health services	Monitoring
	Psychometric test	Analysis of capabilities
	Behavioral observation	Study of behavior in natural environments such as corridors and courtyard
	First trimester screening	Study of the results of the first evaluation
Phase 2, Second quarter	Review of educational measures	Continuous assessment
	Interview with student	Monitoring
	Interview with teachers and head of studies	Monitoring
	Contact with health services	A meeting in mental health services with the student in attendance
	Interview with specialists	Monitoring
	Contact with other external resources	Contact with the specific autism team (this unit also manages psychosis cases). This action involved collecting information within a referral protocol, instruments, and subsequent team meeting
	Behavioral observation	Newly spontaneous behavior was observed
	Second trimester screening	Study of the results of the second evaluation
Phase 3, Third quarter	Interview with student and external resources	The team identified the need for participating in social activities such as “scouts” or workshops (it was not necessary for the new educational therapeutic unit to intervene as it is reserved for cases with greater impact
	Interview with family and external resources	
	Family counseling	Counseling on off-site activities
	Interview with teachers and head of studies	Monitoring and reflection on his academic future
	Interview with specialists	Monitoring and decisions about future support
	Behavioral observation	New spontaneous behavior was observed
	Study of absenteeism	Analysis of assistance throughout the course
	Third trimester screening	Study of the results of the third evaluation. The possibility of including the student in a program to improve learning and performance was postulated. If not possible, the subject shall continue in an ordinary group with support as now
	Interview with family and student	The measure postulated previously was presented to the family
	Development and analysis of memory plans	Reflection on the measures taken to address student diversity
	Closing	Data review

Source: Own elaboration.

**Table 3:** Marks in the three trimesters.

Subject	First Evaluation		Second Evaluation		Third Evaluation	
	Qualitative Rating	Quantitative Rating	Qualitative Rating	Quantitative Rating	Qualitative Rating	Quantitative Rating
Technology	Good	6	Good	6	Good	6
Spanish literature	Insufficient	3	Insufficient	3	Insufficient	3
Physical education (PE)	Sufficient	5	Sufficient	5	Good	6
Math	Good	6	Insufficient	2	Insufficient	3
Biology and geology	Insufficient	4	Insufficient	2	Insufficient	3
Ethical values	Sufficient	5	Notable	7	Sufficient	5
Plastic education	Insufficient	2	Insufficient	3	Insufficient	3
Creation and music expression	Good	6	Good	6	Notable	8
Geography and history	Sufficient	5	Sufficient	5	Sufficient	5
Foreign language	Insufficient	4	Insufficient	2	Insufficient	3
Tutoring	Not evaluable	NQ	Not evaluable	NQ	Not evaluable	NQ

Note. NQ: Not qualified. Source: Own elaboration.

**Table 4:** Measures to manage student educational needs.

General Actions	Description	
**Plan of attention to diversity (PAD)**	General performance	Advice to the educational community (methodology for the programming of subjects) Coordination of all the education agents through Pedagogical Coordination Commission (PCC) Initial interviews with parents
	Ordinary measures	Cooperative learning Coordination meetings with the agents involved Graduation activities Peer tutoring
	Specific measures	Coordination meetings among the agents involved both directly and indirectly in special needs Special attention to the tutor and didactic departments from the guidance department and the school management Organization and implementation of personal resources Specific materials to adapt the curriculum No significant curricular adaptation and significant curricular adaptation if necessary Psycho-pedagogical assessment Personal resources: specialist teacher in therapeutic pedagogy and specialist teacher in audition and language
Tutoring plan	Daily living Social skills activities Emotional intelligence Study strategies Monitoring Reflection on assessment Personal interviews with teachers, relatives, and student	
Academic and career guidance program	Discuss subjects for next year Discuss the educational system Personal interviews with teachers, relatives, and student about future programs	

Source: Own elaboration.

**Table 5:** Legislation by areas.

Area	Rule	Publication in Official Gazette *	Description
National	Organic Law 8, 9 December 2013 [33]	10 December 2013, n. 295, p. 97,858–97,921	Law for the improvement of educational quality
Regional	Decree 359, 30 October 2009 [34]	3 November 2009, n. 254, p. 57,608–57,647	Establishes and regulates the educational response to the diversity of students
	Order of 4 June 2010 [35]	17 June 2010, n. 137, p. 32,839–32,854	Regulates the Plan of Attention to Diversity (PAD)
	Resolution of 3 September 2003 [36]	NL	Provides instructions about the guidance department, which are provided out in secondary schools
	Resolution of 17 December 2012 [37]	22 December 2012, n. 295, p. 51,180–51,189	Guidelines for educational care of pupils with learning difficulties; recently updated (Resolution of 30 July 2019 [38])
	Resolution of 15 June 2015 [39]	24 June 2015, n. 143, p. 25,114–25,117	Establishes who is the recipient of individualized work plans and guidelines for its elaboration
	Resolution of 13 March 2018 [40]	17 March 2018, n. 64, p. 7288–7318	Discusses psycho-educational evaluation
	Resolution of 30 July 2019 [38]	10 August 2019, n. 184, p. 25034-25073	Describes the process of identification and intervention of students with learning difficulties

Note. * BOE or BORM in Spanish depending on the magnitude and influence of the rules. NL: No located. Source: Adapted of BOE for national law and BORM for regional law (Region of Murcia).
